# Complicity or accountability? The limits of positionality statements

**DOI:** 10.1371/journal.pgph.0006042

**Published:** 2026-02-20

**Authors:** Supriya Subramani

**Affiliations:** Sydney Health Ethics, School of Public Health, University of Sydney, Sydney, Australia; Institute of Tropical Medicine: Instituut voor Tropische Geneeskunde, BELGIUM

In recent years, positionality/reflexivity statements have become increasingly common in global health, qualitative health research, and bioethics. Often framed as practices of reflexivity, recently they are also taken up as part of decolonial projects. Yet their growing prevalence invites a critical pause. I argue that thin positionality statements can function less as transformative practices and more as strategies for securing moral innocence, allowing scholars to acknowledge power and privilege without disrupting one’s complicity in retaining them. When reflexivity is reduced to declarations or disclosure rather than accountability, positionality and reflexivity risks becoming a comfort narrative that reproduces, rather than unsettles, coloniality and systemic epistemic and structural injustice. In this paper, I question whether they are normative ‘enough’, particularly in the context of systemic unequal knowledge practices and structural injustice. Specifically, do positionality statements contribute to social justice and decolonial work, or do they perpetuate harm, coloniality and implicate the privileged in imperial and colonial ways of knowledge production? This paper foregrounds complicity as a necessary lens for evaluating positionality statements, rethinking it not as an endpoint of ethical practice but as an ongoing engagement that resists the desire for moral innocence, unsettles privilege, refuses unjust knowledge production and practices, and demands accountability.

## 1. Situating the discourse

I am sure many who work in Global Health, Qualitative Health Research, Bioethics and allied fields have come across positionality statements where researchers list identities and social categories in relation to power, privilege and/or epistemic practices. For instance, these often take the form of: “I am a European, white, cis-gendered male, woman, middle-class, established academic, medical professional, based in the Global North…”or “I am a Brown, Black, cis-gendered woman, working class, economically disadvantaged, queer person of color, based in an LMIC, academic, practitioner, community worker, or public health leader...”.

The calls for reflexivity and reflection on positionality has been increasing, for demand of equitable authorship practices, partnerships and power sharing [1–3], transformation and decolonization of one selves and global health research [4–9]. While scholars have been cautious about the epistemic habits within diverse disciplines [10–14], positionality statements has not yet been sufficiently problematized in global health and bioethics discourse.

Explicit positionality statements have become most common or expected in global health and bioethics scholarship. Researchers now routinely preface one’s work with confessions of racial, gendered, cultural, geographical and able-bodied identity markers—as if such acknowledgements alone render one’s scholarship reflexive and ethically sound. Yet the very proliferation of these statements raises troubling questions: what kinds of mistakes are we making if we assume that locating ourselves in this narrow way is sufficient? And what are the risks when positionality statements become epistemic habits that obscure rather than reveal complicity? In my earlier work [15], I briefly unpacked how narrow or thin reflexivity is practiced within bioethics, stating: “A thin conception of reflexivity is often procedural or instrumental. It acknowledges the need for researchers to reflect on their positionality in narrow statements—mostly, who they are or how they are positioned within social categories—but often limits such reflection to a checklist of considerations, without extending it to theorizing or analysis.” I situated thin and thick reflexivity, and I argued for recognition of the non-innocence of our knowledge production and for the role of reflexivity in demystifying epistemic practices [15,16]. Building on my earlier work, and drawing on the discourse on situated and standpoint epistemologies [17–22], this paper reflects on my recent experiences of attending conferences, workshops, and reading journal articles in global health, qualitative health, and bioethics research. In these spaces, such statements are often invoked—particularly by individuals from relatively privileged groups (for instance, white, cisgender men and women from Global North institutions, or elites in other local contexts, such as upper-caste, upper-class men or women who are aware of their relative power and privilege)—especially in the calls for the decolonization of epistemic practices. My key question is: what does it mean, as epistemic and political practice, to declare, “I am a heterosexual, middle-class, elite, white, able-bodied…”?

When I encounter such statements, my immediate thought is: so, what? Are these confessions, declarations, or disclosures meant to signal privilege? re-emphasize privilege? signal redemption from complicity? To mark the speaker as morally good? politically accountable? or epistemically responsible? Narrow or thin positionality statements often list social categories as separate, additive descriptors, but such framings miss how these identities interact to produce shifting configurations of privilege and marginalisation. Situated epistemologies and intersectionality discourse reminds us that individuals can occupy multiple, layered positions simultaneously. Identities and social categories are social, political and historical positions. A critical account of positionality, as part of reflexive practice, must therefore attend to these intersecting hierarchies rather than rehearsing single-axis descriptions of identity. Often these statements also tend to flatten complexity by implying that individuals are either privileged or oppressed, when often in academic and practice spaces, people inhabit multiple positions simultaneously.

Positionality is never singular. For instance, one may occupy racial and institutional privilege while being gender-marginalised; experience racial marginalisation alongside gendered authority; inhabit colonial precarity within global knowledge economies while exercising caste, class, or gender dominance locally; or be queer and working-class yet wield institutional privilege as a Global North academic. These configurations do not cancel one another out; rather, they coexist, shift across contexts, and demand ongoing ethical attention to how power is exercised, not merely how identity is declared. These layered configurations shape how authority, credibility, and vulnerability are distributed in any given situation and relationships. When these complexities are reduced to a checklist of identity markers, positionality becomes a descriptive exercise rather than an analytical one, obscuring how privilege is reproduced across intersecting structures of power. Also, it shifts across geopolitical, institutional, and interpersonal contexts. These contextual shifts matter because narrow positionality statements often present identity as static, masking how power is contingent, situational, and relational. Without attending to how privilege and marginalisation recalibrate across settings, positionality statements risk reinforcing a simplistic narrative that “this is who I am,” rather than asking “how do my relations of power change in this context, and what does accountability require here?” Here, building on earlier work and my own continued unlearning’s, I critique practices that reduce reflexivity to positionality statements while evading its political and ethical commitments.

My aim in this paper is not to rehearse the now well-established arguments for why social categories matter for knowledge practices—arguments that critical and feminist and social epistemologies have made them visible [17,18,20,23,24]. However, I interrogate what these statements, confessions or declarations are doing and why we need to critically reflect on them as practices that must be situated within systemic injustice, within both knowledge practice and politics. I argue that when positionality statements are presented without this deeper engagement, they risk functioning as complicity—as unfair knowledge practices perpetuated in the name of morality and sometimes with the best of intentions and they become mere performative epistemic habits. Then it functions as rhetorical, deflective and protective tools. And they reproduce a politics of benevolence—where being a “morally good person” becomes part of the problem rather than the solution to epistemic and structural injustice. This paper therefore asks what it means to write or declare positionality statements when one is privileged and illustrate how complicity as a necessary lens for evaluating narrow positionality statements.

In the next sections, I first briefly reflect on positionality in relation to larger discourse, and specifically to global health and bioethics, followed by illustrations through analytical modes. These modes are not exhaustive, but they show how privilege is acknowledged while complicity remains untroubled.

## 2. Reflections on Positionality

For scholars who have experienced historical and ongoing injustice and violence in all its varied forms, being reflexive means recognizing political identities and their ramifications in, on, and with our research and practice at each stage. While different arguments and engagements are necessary when scholars who experience disadvantage, oppression, and violence are researching and employing reflexivity [17,18,20], critical scholarship shows us how they often center and disrupt institutions and reflect on insider/outsider/foreign contexts and situations. Furthermore, many scholars of situated and indigenous epistemologies reflect on their intersecting identities and situate those identities to *contextualise* one’s theory and practice, rather than as a claim to ‘epistemic authority’ [17,22,25–28].

Critical scholars argue for rejecting ideals of objectivity and recognizing that our identities—constructed and diverse—and our positionalities influence the process of knowledge production, making it essential to critically reflect on them [20,22,26,29,30]. Learning from scholars who embrace the situated epistemologies [17,18,20,24,29], where researchers must recognize and take responsibility for how our identities influence the research we conduct and produce—I take forward and acknowledge the ethical and political ramifications of reflexivity [15], where reflections on positionality are one among many reflexive methodological practices. For those familiar with the worldview that knowledge production is inherently political, there is a strong awareness of power dynamics in all its forms, whether as epistemologies of ignorance or epistemologies of awareness. Recognizing one’s positionality in knowledge production assumes a particular ontological worldview that intertwines ethics, embodiment, and politics in understanding social reality. For instance, in my earlier work [31], I attempt to show how engaging with concepts such as cosmopolitanism demands that we critique the disembodied notion of moral equality in the current narrow visions and calls for cosmopolitanism in public health ethics and migration health—visions that masquerade as universal, non-historical, and difference-blind.

Doing and practicing global health/bioethics research in a world of violence—amidst ongoing genocide in Gaza, military invasion, political violence, settler-colonization, increasing securitization and territorialization, and post-war settings where humanitarian needs, displacement, and refugee camps are prevalent—is not “normal.” These research and practice settings in global health and global bioethics scholarship are often marked by colonial or imperial histories and relationships. This legacy creates intricate geopolitical dynamics, persistent inequalities, and demands collective reflection from researchers and practitioners. Against the backdrop of increasing calls for decolonization and inclusion in global health and bioethics research [4,6,32–34], where power has been central to these discussions [35,36], some scholars have begun considering the positionality of Global North scholars in relation to the shifting burdens placed on Global South scholars to draw attention to injustices in academic scholarship and knowledge production [37]. Meanwhile, for example, a few Global North scholars, routinely include a short positionality statements (e.g., positioning themselves in relation to authorship conversations [38]), while journals propose reflexivity statements for equity purposes [3].

In these statements, one should pay close attention to spatial-historical location—becoming aware of one’s complicity in ongoing global health imperialism and colonialism, which involves appreciating both one’s locations and one’s limits. Being well aware of White saviorism and Brown saviorism in global health and ethics spaces [39], it becomes evident that we are implicated. As Ahmed suggests, “we are implicated in the worlds that we critique; being critical does not suspend any such implication” [40], and assuming one’s criticality can itself be a way of not admitting one’s complicity. Complicity, then, is a starting point to address epistemic and structural injustice, and it demands vigilance and accountability from those who are privileged. In practice, this sometimes means not doing the research at all or stepping back from positions that sustain unjust norms and structures; at other times, it means doing the work through complete reimagination, drawing from anti-colonial and decolonial ways. Global health researchers are—or should be—aware of the power asymmetries inherent in such contexts, including how we study, write, engage, and present, whether as normative topics or as people’s stories, narratives, and experiences [41,42].

The Lancet Global Health [43] editorial acknowledges that imbalances in authorship are symptoms of broader imbalances of power, and it calls for greater “editorial reflexivity” and attention to who tells the story in global health. Scholars pushes this critique further by naming the persistence of “Northern ventriloquism,” whereby LMIC scholars reproduce the epistemologies of the Global North in order to be heard, and by urging practices of “epistemic disobedience” that resist such silencing [8,14,37,44]. Both interventions highlight how reflexivity, when framed narrowly, risks becoming an exercise in re-centring the Global North subject rather than dismantling structural hierarchies.

Reflecting on ourselves, as both recognition and resistance, narrow positionality statements—as currently practiced—are a critical moment of pause to ask whether they move us toward accountability or instead reproduce complicity. Writing these narrow positionality statements in global health and ethics research, especially for those who hold power and privilege due to class, cultural, racial, gender, location and political factors, often serves not only as a methodological tool but also as virtue signalling within current debates on decolonization. Turning the gaze toward those who hold power while engaging with positionality statements demonstrates two key aspects: attitudes and emotions, and our/their relationship to identities, knowledge, and political practices. In the next section, I will unpack complicity as a necessary lens for evaluating positionality statements.

## 3. Ethical Shortcuts: The moral work of positionality statements

As noted earlier, privilege and power are always occupied relationally and in relative terms, shifting across contexts, institutions, and social locations. Yet within global health and bioethics, race—and whiteness in particular—remains conspicuously under-engaged, even as other axes of difference are more readily acknowledged. This silence is not incidental. Rather, it reflects the political neutralisation and disciplinary co-optation of positionality statements, mirroring the assimilation of intersectionality into procedural checklists that leave underlying hierarchies intact. While recognising the intersecting operations of class, religion, gender, sexuality, ableism, caste and citizenship, this section therefore focuses analytically on whiteness as one critical axis through which to unpack how ethical shortcuts are reproduced within contemporary imperial and colonial disciplines such as global health and bioethics.

In today’s unequal and unjust global order—marked by deep hierarchies that are mirrored in academic disciplines such as global health and bioethics—whiteness remains central to the production and maintenance of injustice. At the same time, scholarship and practice often normalise an exclusive gaze on the racialised “Other” under the banner of “help,” “rescue,” or “development.” Consider a racially and class-privileged scholar—let us call them Hotspot. When Hotspot declares, “I am a white, cis-gendered male in a Global North institution,” against this backdrop of entrenched inequality and white supremacy, the statement is rarely innocent. Rather, it performs particular functions: it may signal acknowledgement of privilege, stage a performance of reflexivity, or operate as a confession intended to secure moral innocence. Some may respond to racial critique through defensive moves that re-centre innocence or discomfort—forms of white fragility and ignorance that help contain rather than confront power.

Before turning to the four analytical modes, it is worth pausing on why such categories matter. Analytical categories or modes are a like carnival mirrors: they do not create the distortions, but they help us notice them. By giving ‘Hotspot’ a name and tracing their tendencies, I am not inventing a caricature but holding up a mirror to familiar patterns that appear in global health and bioethics writing, scholarship and practices. And categories are not just descriptive; they are diagnostic tools that reveal the moral work of positionality/reflexivity statements. They allow us to see how declarations of identity are never innocent: they are entangled in structures of power, bound up in desires for moral innocence or resistance, and often implicated in what they claim to resist. In this sense, categories sharpen our vigilance by showing how easily positionality and reflexivity collapses into performance, confession into redemption, or solidarity into self-protection.

With this in mind, I outline four recurring, and sometimes overlapping, modes through which positionality statements authored by racially privileged scholars operate. These modes are not exhaustive — Hotspot has many disguises — but they capture some of the key moves through which power and privilege, in this example whiteness, is acknowledged while complicity remains untroubled.

### 3.1. Acknowledgment as admission

Hotspot may openly admit their privilege and power, situating themselves as racially and structurally advantaged. This can serve as a necessary recognition that academic disciplines are not neutral. Yet, acknowledgment often stops at naming — it risks being a declaration that signals awareness while leaving untouched the deeper entanglements through which privilege is sustained. In this mode, complicity remains unaddressed, hidden under the appearance of acknowledgment.

### 3.2. Reflexivity as performance

Another common move is virtue-signalling, where positionality is mobilized to present oneself as “aware,” “reflexive,” or even “decolonial.” Hotspot’s statement, then, is less about transforming practices and more about curating a scholarly persona that demonstrates “goodness”. The danger here is that it is reduced to performance — a badge of credibility that allows scholars to appear accountable while avoiding the difficult work of unsettling structural hierarchies.

### 3.3. Confession as redemption

In this mode, Hotspot deploys positionality as a confessional exercise, listing privileges, in order to symbolically “cleanse” themselves. Confession becomes a way of securing moral innocence: once declared, complicity is paradoxically disavowed. Such utterances often do the opposite of what they claim — in saying “I am complicit,” the speaker implies “I am now innocent.” Rather than unsettling whiteness, confession can re-center the privileged self as the locus of moral action.

### 3.4. Solidarity as innocence

Finally, Hotspot may frame positionality through declarations of partnership or solidarity with the marginalized. While often motivated by good intentions, such gestures risk masking asymmetries in authorship, funding, and agenda-setting. The claim to “stand with” others can function as a shield, portraying the privileged scholar as ethically aligned while concealing the structural conditions that reproduce inequality. In this way, solidarity narratives may re-inscribe Global North centrality even as they profess to decentre it.

Taken together, these modes show that positionality statements are not neutral reflections but political acts. The analytic challenge, then, is to recognize how these statements may operate less as transformative practices and more as modes of moral innocence. Also, the problem with these accounts of statements is that they risk centring the individual while leaving the system of epistemic injustice and global inequalities substantially unchallenged. Focusing on whether Hotspot “acknowledged privilege” or “signalled awareness” risks reducing to individual behavior, leaving intact the very system that privileges certain kinds of knowledge, methodologies, and institutional actors over others.

If whiteness and white supremacy are at the heart of injustice, does it mean then all those belong to these categories are complicit? Then what does it mean to acknowledge power and privilege due to one’s embodied and institutional interlocking privileges? Does it mean then privileged can’t be escaped of structures of domination and injustice? Seen in this light, narrow positionality statements from privileged scholars risk becoming an ethical shortcut: by naming one’s positionality, the speaker performs acknowledgement but stops short of grappling with what that acknowledgement entails. To confess is not the same as to disrupt. The deeper challenge lies in refusing the comfort of confession and asking instead: how does one’s scholarship, institutional participation, and everyday practice actively reproduce or resist the interlocking systems of epistemic injustice and global health inequalities?

The point, however, is not whether the author of a positionality statement is a “good” or “bad” person. Framing the question in this way only re-centers the interests, anxieties, and needs of privileged scholars rather than attending to the structural inequalities and epistemic harms and violences experienced by marginalized communities. Narrow positionality statements—those limited to brief declarations of privilege, identity, or confession—often evade these questions. By focusing on naming oneself (e.g., “I am X, Y, Z”), such statements risk reducing structural complicity to an individual performance of reflexivity.

A more critical orientation would shift from confession to action: not simply what identities do I hold. But how do these identities, embedded within a global system of inequality, shape what kinds of knowledge we are able to produce, circulate, or silence. If we focus on ‘what is it doing?’ It becomes necessary to address colonial, imperial, and racist systems, relationships and institutions which normalise power asymmetry by suggesting ‘this is the way it is’ and ‘we are all just helpless’. I will focus in the next section on the dimension of privilege, which is central to this analysis: how thin positionality statements by the privileged become problematic, serving less as tools of decoloniality and accountability and more as practices that retain coloniality and reproduce privilege, and specifically whiteness in relation to racial privilege and practices.

## 4. Unsettling privilege

The language of privilege has played an important role in shifting conversations away from individual prejudice toward the broader, systemic advantages that flow to members of dominant groups [45–48]. These benefits are not earned but attached to social position, often irrespective of personal intention, belief, or attitude. Often, as a ripple effect, across disciplines, a central strategy for those privileged to their place within structures of racial injustice has been what is often called white privilege which designed to prompt recognition and acknowledgment of one’s own racial privilege. On this account, reading the declarations or confessions, then can be seen as moment of “change” where we have “arrived” to acknowledge power and privilege. But failing to recognize the hidden structures of advantage means not only ignoring their existence but also reproducing them.

Privilege is not distributed in identical ways; due to intersectional categories one embodies. And too often, such statements reduce complex histories and structures of inequality to overly simplistic declarations of identity and awareness. By centering declarations of privilege as if they were self-contained, they risk portraying privilege as an individual possession rather than as a relational condition produced through histories of colonialism, exploitation, and epistemic exclusion. Privilege, whether material or epistemic, is always dependent on the simultaneous marginalization of others. To name privilege without interrogating this interdependence reduces positionality to confession, while leaving untouched the unjust structures through which such privilege is sustained. Much like the idea that one can “take off” the knapsack of privilege, positionality statements can create the impression that naming one’s privilege or declaring one’s location is sufficient to divest oneself of complicity. The implicit promise is that through confession, a purified or neutral subject can emerge—one who has acknowledged their advantages and is therefore absolved.

This understanding reduces decolonisation or the addressing of injustice to a personal act of declaration. It overlooks how privilege is not detachable from the constitution of the self, nor separable from the systems of knowledge, institutions, and histories through which global health research or global bioethics is organized. Moreover, it obscures how discourses of reflexivity and confession can themselves become mechanisms of protection—shielding systems of inequality from deeper contestation by allowing privileged actors to appear accountable while leaving the underlying structures of coloniality, imperial structures, and systemic injustice intact.

Finally, and closely linked to the problem of individualism, positionality statements in global health risk becoming a form of academic confession. More troublingly, confessional positionality can allow privileged actors to displace attention away from how their scholarship, institutional affiliations, and everyday practices actively reproduce global health inequalities and knowledge practices. In other words, by confessing, one can appear reflexive while avoiding deeper accountability for complicity in maintaining the very systems of domination that positionality statements claim to confront. Thus, even when positionality statements openly acknowledge complicity, they can function as academic confessionals that reinscribe privilege. In these moments, positionality risks resembling a practice of penance: difficult truths are spoken aloud, but the act of disclosure itself becomes the redemptive gesture. This dynamic is particularly pernicious, as it allows privileged scholars to appear reflexive or decolonial while avoiding engagement with how our knowledge production, institutional affiliations, and professional practices continue to reproduce coloniality and epistemic injustice. The critical task, then, is to foreground not only the naming of privilege but also how such privilege actively sustains global health inequalities and demands accountability beyond narrow positionality statements. One way to see this moment as an opportunity to make explicit the connection between the privileges that benefit scholars in positions of power and the ways those very benefits are bound up with sustaining systems of epistemic injustice and global health inequality.

## 5. Privilege, Ignorance, and Complicity

It is the structures of coloniality and white supremacy that make academic and professional privilege possible for those racially privileged (white privilege in specific, [46–49]). Then, white privilege is not simply a matter of passive benefit; it involves the ongoing extraction of resources, the appropriation of labor, and the construction of policies and practices that systematically deny marginalized communities’ full participation [47,49]. To ignore these active processes is to perpetuate the myth of white innocence. Then the critical account of white supremacy or conditions which makes privilege possible needs to be understood, and attend to the direct processes that secure domination and sustain privilege [46]. In global health and bioethics, this means recognizing that privilege is not simply “held” by individual scholars and institutions but continually produced through research agendas, funding priorities, partnerships, and publishing practices that extract from and marginalize others.

If privilege is produced, then, I would argue, thin positionality statements on privilege and power function in ways strikingly similar to what critical scholars have described as the whitening or neutralisation of intersectionality [50,51]. For instance, this neutralising effect is particularly visible in humanitarian and migrant health discourses, where appeals to universality and neutrality coexist uneasily with a rapid rhetorical embrace of decolonial language and frameworks, and whiteness and white supremacy is absent. Much like intersectionality’s institutional uptake, positionality is increasingly rendered as a procedural requirement rather than as an epistemic disruption or a form of epistemic and political accountability to the communities one works with. This is particularly salient in fields such as global health and bioethics, which continue to be organised around dominant Eurocentric epistemologies and practices while selectively incorporating critical vocabularies. These disciplines, among others, have historically sustained one of the most enduring myths of Eurocentrism: the myth of a neutral, universal, and objective point of view.

In these fields, claims to universality and objectivity routinely marginalise forms of knowledge that are affective, situated, embodied, or politically explicit, casting them as anecdotal, subjective, or insufficiently rigorous. As a result, epistemologies grounded in lived experience—particularly those emerging from racialised, colonised, migrant, or minority communities—are often tolerated only as illustrative material rather than as sources of theory, critique, or normative authority. The decolonial challenge, then, is not simply to diversify who speaks or which identities are named, but to remain alert to how whitening forces operate across disciplines—absorbing critique without unsettling the epistemic and political structures that sustain authority, legitimacy, and governance. Thus, thin positionality statements, in this context, help stabilise the field’s self-image as critically aware while deflecting attention from its complicity in neoliberal and colonial modes of knowledge production.

What is required is an approach that foregrounds complexity and insists that those who benefit from privilege remain accountable for how it is reproduced. Privilege is not only about access to resources and opportunities; it also enables forms of ignorance, arrogance, and obliviousness that allow injustice to persist unchecked. This negative privilege is often enacted through discursive practices that deny complicity while maintaining a posture of innocence. To ignore such manifestations of privilege is to disregard the ways in which well-intentioned, seemingly progressive actors continue to sustain injustice in the present. Thin positionality statements often highlight the positive dimensions of privilege—identities, resources, access, authority—without equal attention to these negative dimensions: the ways privilege permits actors to avoid accountability, responsibility, to re-center themselves, and to reproduce hierarchies under the banner of reflexivity or decolonisation. When framed this way, positionality becomes less a tool of accountability and more a mechanism for protecting innocence. It allows those who benefit most from global health inequalities—whether in the Global North or among elites in the Global South—to appear reflexive while continuing to exercise dominance.

Negative privilege manifests when privileged scholars assume the right to define research agendas, move seamlessly across communities and contexts, and claim authority over knowledge without recognizing how their practices displace communities’ ideas and experiences. For example, if we look at some global health and bioethics works when talking about migration health, the “migrant crisis” is decontextualized and disembodied through abstract concepts and through one’s language of obscurity [31]. When scholars abstract and decontextualize, the risk is of political neutrality. There is a tight link between privilege and ignorance, Mayo [52] reminding us of that ignorance itself can be systemic. Further, Charles Mills [53] through his account of an epistemology of ignorance—a socially functional set of cognitive habits that keeps privilege invisible to those who benefit from it. Mills poses the question: How is it that white people can so consistently do the wrong thing while believing they are doing the right thing? His answer is that white ignorance is not simply a matter of personal failure but a patterned epistemic practice that produces the ironic outcome of rendering whites unable to grasp the world they themselves have structured. DiAngelo’s [54] analysis of white fragility can also be considered here, for instance, in showing how reflexive gestures can function less as openings to ethical accountability and more as mechanisms of deflection and containment. And, Linda Alcoff [55,56] similarly cautions against reducing white ignorance to poor individual judgment; rather, it must be understood as an organized epistemic practice in its own right.

In The Racial Contract [57], Mills argues that this ignorance underwrites modern social and political life. The Racial Contract is a tacit agreement among whites to create and maintain a subperson class of non-whites, thereby securing the privileges and advantages of full white citizenship. To sustain this arrangement, ignorance must be cultivated, an agreement “not to know”, which is then legitimated as a true and authoritative version of reality. Crucially, this ignorance does not merely persist despite social systems but is actively sanctioned by them, such that what is in fact misinterpretation appears as knowledge. This analysis has sharp implications for us. When privilege is made visible only through narrow positionality statements, the deeper epistemology of ignorance remains intact. The systemic production of not-knowing—about colonial histories, about the extraction of labor and resources, about the epistemic authority granted to Northern institutions and Southern elites—continues to operate as if it were knowledge. Positionality statements that stop at confession risk reinforcing this very dynamic: they allow privileged scholars to appear reflexive while leaving the sanctioned ignorance that sustains inequalities unchallenged.

Mayo’s point becomes clearer when read alongside Mills’s observation that ignorance shapes not only what people believe, but also what they regard as worth asking and pursuing. For instance, “migration crisis” or migrant health ethics discourse conceals the coloniality and imperialism, capitalism and geopolitical conditions that drive dispossession and displacement, and often “climate crisis” distances the capitalistic, colonial and imperial conditions that exploit nature and normalize inequalities, and interventions often target individual behaviour, attitude or actions. Ahistorical, apolitical, and decontextualized representations are common in dominant bioethics and global health discourse, and it produces abstract decontextualised moral knowledge about ‘Others’ that legitimize and sustain the power asymmetries. However, given the often-narrow epistemic habits and moral signalling of positionality statements, it becomes evident to engage with its significance.

Ignorance itself thus operates as a form of privilege—who, after all, has the privilege to remain ignorant? Mills underscores that privileged group’s interest is central to producing and sustaining this ignorance, which allows beneficiaries of unjust systems to mystify their own complicity. Members of dominant groups not only have less incentive to understand systemic injustice than those who endure it, but they often have a positive interest in “not knowing” [55]. Such ignorance serves to sustain one’s moral self-image: to remain innocent, even while implicated. In global health and bioethics, we can see reflections of this and how it is mirrored in the ways positionality statements are often mobilized. By stopping at acknowledgement of privilege, they may obscure the deeper questions that urgently need to be asked: How are research agendas shaped by colonial histories? Whose knowledge and methods are deemed legitimate? Who is excluded from authorship, funding, or institutional recognition? Narrow confessions of privilege, when detached from these systemic inquiries, function as another form of sanctioned ignorance—allowing scholars to preserve their moral self-image while leaving global inequalities untouched.

## 6. Toward Reflexivity that Demands Accountability

Producing knowledge is a political endeavor that can reinforce dominant interests and justify violence, consciously or subconsciously [12,13,18]. Often privileged researchers, through academic institutions and epistemological frameworks, enable and facilitate colonial and imperial projects in the name of the ‘best interest’ and for the good of ‘distant poor others’ or close ‘Others.’ For positionality statements to be effective and contribute to decolonial efforts in current global health and global bioethics discourse, we/they must first acknowledge researchers’ complicity within imperial, racial, and colonial power structures and knowledge production. If researchers fail to consider one’s politics of location and the broader historical and ongoing injustices when studying marginalized voices and collaborating with researchers and communities from these contexts, we/they are complicit in adopting a narrow version of positionality and reflexive practices. This narrow approach normalizes and takes the status quo for granted, ultimately benefiting the privileged. If researchers briefly acknowledge their positionality but do not actively engage with marginalized voices or center marginalized scholars and don’t practice accountability to undo these structures, we/they perpetuate the system that maintains and reinforces power and knowledge imbalances. When scholars critically recognize that they cannot address systemic issues but still focus solely on their positionality without engaging with the political and ethical implications of their research practices, it is crucial to confront this. And then we must ask ourselves why we do this exercise, for whom, and what role we play in complicity.

Does this mean, then, that it is inevitable for dominant and privileged groups to sustain injustice by default, and that positionality statements hold no significance within these unjust systems? Does this mean we should not engage with positionality at all? Just because someone is heterosexual and white does not mean this person inevitably cannot be or not feminist or anti-racist. This understanding demands that there is a certain reasoning involved when we declare our identities. But to practice positionality and reflexivity is to critically ask what it is serving and for whom. It also demands that we take responsibility and hold ourselves accountable. That sometimes means having the humility to acknowledge when it is best to step back, give up positions of power, check our social and emotional needs for being “benevolent,” or instead institute structures and processes that facilitate meaningful and respectful engagement, where the marginalized can have voice, power, and resources. It also means consciously addressing unjust structures that retain hierarchies, and engaging, listening, and learning from communities who are already doing this work outside academia and other spaces. Above all, this practice—and those who engage in it—should be accountable and responsible in ways that decenter the conversation in relation to one’s own work and epistemic practices, rather than reinforcing unjust systems under the banner of signalling and being ‘good.’ All of this means putting ourselves in discomfort, recognizing how we are complicit in the system, and working toward undoing systemic structural injustice.

A growing body of scholarship has examined how denials of complicity function discursively to protect privilege. These denials allow privileged to distance themselves from responsibility for systemic injustice while maintaining a sense of moral innocence. Positionality statements, when framed narrowly as self-disclosures, can play a similar role. By presenting acknowledgment as sufficient, they create space for privileged actors to appear accountable while avoiding deeper engagement with how their practices sustain structural inequities. In my own teaching of public health ethics in a white-dominant university (though not always in white-dominant classrooms), where I insist on reflexive practice as central, these dynamics are evident. When positionality enters classroom discussions, some racially privileged students respond with visible defensiveness or disengagement. Comments such as, “Why is it always about racism and colonialism—can’t we do public health ethics without it?” or “I am white, so people from LMICs, tell me about your experiences,” reveal how racially privileged students reflect on their identities—or choose not to. Others retreat behind claims like, “I can’t say anything these days,” or adopt distancing behaviors that shield them from participating. In professional contexts, I have encountered similar reactions. At conferences, some white scholars have remarked: “I know I am white, but what can I do? I can’t change my skin,” or “It is easier for you to speak about reflexivity because you are brown.” These statements mirror the very logic of positionality confessionals: privilege is acknowledged but simultaneously recast as an immutable fact that exempts the speaker from both accountability and responsibility.

As Peggy McIntosh [47] suggests, privilege grants “permission to escape,” and Alice McIntyre [58] names it a “privileged choice”: the ability to disengage from uncomfortable conversations without consequence. I would argue, this is also how positionality statements can function. When framed as mere confessionals or declarations, they risk becoming a form of white talk—a discursive strategy that insulates the privileged from interrogation. Declaring, “I am white, therefore I acknowledge my privilege,” or “I cannot change my skin,” can serve as a performative endpoint rather than an opening for accountability and responsibility. The statement itself becomes the evidence of one’s goodness, restoring a sense of moral standing without requiring structural critique. In global health and bioethics, positionality statements can operate in precisely this way. They promise accountability but risk delivering protection: protection of innocence, protection of institutional legitimacy, and protection of privilege itself. By centering confession rather than complicity, they obscure how privilege is continually reproduced through research agendas, funding structures, authorship hierarchies, and epistemic exclusions. The danger, then, is not only that positionality statements fall short of dismantling coloniality, but that they actively participate in its reproduction.

The implication of my arguments is this: once I acknowledge my complicity and reject any notion of my own innocence, benevolence, or value-neutral understanding of research—as well as my inability to transcend the systems of domination in which I am entangled—various assertions become contentious. Viewing these statements through the perspective of complicity imposes upon us a dual moral obligation: politically, we must address and challenge the systems of domination of which we are a part; intellectually, we must strive to understand these systems, their functions, and their consequences, and centering marginalized voices. This means I cannot make claims about doing global health research on “distant poor others” or “Others,” their best interests, or their futures. Such understanding must come from intentional and meaningful interactions with those who possess knowledge, through listening to stories and experiences that we lack. It is not sufficient to merely acknowledge our positionality or gatekeep positionality statements as equitable authorship practices alone; we must also recognize that, as privileged group in academia, we are inherently engaged in political and epistemic relationships that are predominantly problematic under the banner of “research”, and we must attempt to address and undo the structures that uphold power.

Recognizing complicity then demands us to question the very structures of research process which has colonial and imperial logics, particularly in fields like global health and bioethics, and refuse to be part of it. In social and behavioural research across these disciplines, particularly among those of us who seek to do—or claim to do—decolonial work, Eve Tuck and K. Wayne Yang’s [59] call to refusal is instructive. They urge a refusal to become a “voicebox, ventriloquist, or interpreter of subaltern voice,” and a refusal to act as a “purveyor of voices”. I would see then refusal, in this sense, is not methodological withdrawal but ethical accountability and responsibility. It includes refusing to write pain narratives and stories for the consumption of privileged gazes and the logics of dominant knowledge systems. It also requires allowing ourselves to question the deeply entrenched assumption that research is fundamentally good for our communities, particularly when we already know that there are far more instances than are commonly acknowledged in which research is neither the most useful nor the most appropriate intervention. This refusal demands a departure from knowledge practices designed for elite scholarly consumption—practices that remain disconnected from the communities we work with while catering to a privileged gaze. For those of us who occupy relative privilege while working with our communities, refusal demands that we ask, as Simpson [25] does: Can I do this and still come home; what am I revealing here and why? Where will this get us? Who benefits from this and why? To this, I add a further set of questions that refusal compels us to confront: for whom am I writing, and why? For what political and ethical commitments? What does it mean to authentically work with communities without assuming that we are the voice, that research is the answer, or that representation is the primary form of intervention? Refusal, then, opens up the ethical and political necessity of finding creative, relational, and non-extractive ways to intervene and engage. And it becomes a demand for accountability rather than declaration, refusing the comfort of positional self-disclosure and insisting instead that epistemic and political commitments be enacted through practices that actively confront complicity, unsettle privilege, and disrupt the structures that grant us “epistemic authority”.

Thus, to summarise, when scholars write positionality statements such as, “I am a heterosexual, middle-class, elite, white, able-bodied male or female,” or “anyone who identifies as X, Y, Z” without practicing accountability within our work and everyday practice, such declarations risk recentering privilege and illustrate the denial of complicity in systemic injustice. Reduced to a list of identity markers, these statements can function as confessional rituals—performances of awareness that end with self-disclosure rather than accountability. Instead of unsettling privilege, they often recenter the privileged subject, making the declaration itself evidence of being a “good,” “aware,” or “decolonial” scholar. This kind of virtue signaling risks relieving guilt without demanding structural change. There is no moral innocence; we can only be accountable and responsible. Acknowledgment of privilege without confronting complicity and addressing it becomes a denial of accountability and responsibility. In academic domains like global health and bioethics, such flat disclosures depoliticize reflexivity by stripping it of historical, material, economic, political, and systemic injustices: we say, “this is who I am,” without asking, “how are we implicated, how do we benefit, and how and what will we do differently?” In this way, thin positionality statements and practices can perpetuate the very privilege, imperiality, and coloniality they claim to resist, rather than advancing decolonial efforts within epistemic practices. Against epistemic and structural injustice in global health and bioethics—and the limitations of thin positionality statements—holding power and privilege therefore demand accountability. Thus, positionality statements can only be meaningful if it does more than confess location; one must make visible the action-based practices through which scholars are willing to unsettle and undo the power relations that sustain our authority. Without this shift from acknowledgment to accountability, it risks becoming another neutralising gesture—reflective in appearance, yet politically inert.
